# Synergistic Effect of ZIF-8 and Pt-Functionalized NiO/In_2_O_3_ Hollow Nanofibers for Highly Sensitive Detection of Formaldehyde

**DOI:** 10.3390/nano14100841

**Published:** 2024-05-10

**Authors:** Lei Zhu, Ze Wang, Jianan Wang, Jianwei Liu, Wei Zhao, Jiaxin Zhang, Wei Yan

**Affiliations:** 1 Xi’an Key Laboratory of Solid Waste Resource Regeneration and Recycling, State Key Laboratory of Multiphase Flow Engineering, School of Energy and Power Engineering, Xi’an Jiaotong University, Xi’an 710049, China; leizhu@xjtu.edu.cn (L.Z.);; 2 School of Physics and Electrical Engineering, Weinan Normal University, Chaoyang Street, Weinan 714099, China; 3 Xianggui Manganese Industry Co., Ltd., Ziyang, Ankang 725300, China; 4 School of Chemistry and Chemical Engineering, Xi’an University of Science & Technology, Xi’an 710054, China

**Keywords:** formaldehyde sensing, p-n heterojunction, Pt catalysts, ZIF-8 nanoparticles

## Abstract

A rapid and accurate monitoring of hazardous formaldehyde (HCHO) gas is extremely essential for health protection. However, the high-power consumption and humidity interference still hinder the application of HCHO gas sensors. Hence, zeolitic imidazolate framework-8 (ZIF-8)-loaded Pt-NiO/In_2_O_3_ hollow nanofibers (ZPNiIn HNFs) were designed via the electrospinning technique followed by hydrothermal treatment, aiming to enable a synergistic advantage of the surface modification and the construction of a p-n heterostructure to improve the sensing performance of the HCHO gas sensor. The ZPNiIn HNF sensor has a response value of 52.8 to 100 ppm HCHO, a nearly 4-fold enhancement over a pristine In_2_O_3_ sensor, at a moderately low temperature of 180 °C, along with rapid response/recovery speed (8/17 s) and excellent humidity tolerance. These enhanced sensing properties can be attributed to the Pt catalysts boosting the catalytic activity, the p-n heterojunctions facilitating the chemical reaction, and the appropriate ZIF-8 loading providing a hydrophobic surface. Our research presents an effective sensing material design strategy for inspiring the development of cost-effective sensors for the accurate detection of indoor HCHO hazardous gas.

## 1. Introduction

Formaldehyde (HCHO) is regarded as a common indoor pollutant. Prolonged exposure to high levels of HCHO can lead to a variety of health problems [1,2,3]. The World Health Organization (WHO) guidelines have set an exposure threshold value of 82 ppb for HCHO in indoor living areas [4]. At present, metal oxide semiconductor (MOS)-based sensors are one of the effective methods for monitoring indoor HCHO with higher sensitivity and shorter reaction times [5,6]. However, the high-power consumption of MOS sensors and the susceptibility of their sensitivity to ambient humidity are the main drawbacks that affect the reliability of gas sensors in practical applications [7]. Thus, it is necessary to develop an MOS-based sensor with a lower operating temperature (<200 °C) and good anti-humidity for high-sensitivity HCHO detection.

The surface modification of MOS structures by noble metals (Pt, Ag, and Pd) with unique electronic states and chemical properties reduces the adsorption activation energy and promotes the electron transfer efficiency, thus enhancing the sensing capabilities of MOS [8]. For instance, Liu et al. [9] synthesized Ag-SnO_2_ nanoparticles, and the sensitivity reached 14.4 towards 10 ppm HCHO at 125 °C. In addition, p-n heterojunctions can integrate the physical and chemical properties of p- and n-type MOS into one system [2,10,11]. Nickel oxide (NiO), as a typical p-type metal oxide, can accelerate the catalytic decomposition of the target gas and promote the gas-sensing reaction on the surface [12,13], further improving the response/recovery characteristics and reducing the operating temperature of the gas sensor. Kumar et al. [14] prepared SnO_2_-decorated NiO nanoparticles and investigated the catalytic kinetics during the HCHO adsorption–desorption process. As mentioned above, the construction of hybrid materials combining noble metals and p-n heterojunctions is expected to realize the highly sensitive detection of HCHO gas at low temperatures.

It has been suggested that the surface modification of MOS materials can improve the water-resistance performance of gas sensors by ensuring minimal effects from humidity [15,16,17]. Metal–organic frameworks (MOFs) featuring a high specific surface area and abundant porous structure can effectively avoid contact between water molecules and MOS-sensing materials [18]. Meanwhile, gas molecules can diffuse through the pores of MOFs [19]. Hence, MOF-based hydrophobic surface modifications have been found to enhance the anti-humidity performance of MOS-based gas sensors without reducing their sensitivity. 

Zeolitic imidazolate framework-8 (ZIF-8), a well-studied MOF with a robust structure and highly porous properties, is composed of Zn^2+^ ions coordinated with 2-methylimidazolate ligands, which has a wide range of applications in gas sensors. In addition, the hydrothermal technique is a facile and effective method for the synthesis of gas-sensing materials. Ferlazzo et al. [20] prepared samarium oxide (Sm_2_O_3_) nanorods by a facile hydrothermal route for efficient detection of volatile organic compounds gas in the indoor environment. Jin et al. [21] constructed SnO_2_/SnS_2_ n-n heterojunction anchored on rGO using a hydrothermal route for detection HCHO.

Herein, ZIF-8 loaded on the surface of Pt-NiO/In_2_O_3_ hollow nanofibers (ZPNiIn HNFs) were synthesized by facile electrospinning technique combined with the subsequent hydrothermal method to serve as the high-performance HCHO gas-sensing materials. To evaluate the effect of p-n NiO/In_2_O_3_ heterojunctions, ZIF-8, and noble metal Pt nanoparticles on sensor performance, HCHO-sensing measurements based on pristine In_2_O_3_, NiO/In_2_O_3_, ZIF-8@NiO/In_2_O_3_, and ZPNiIn sensors were carried out. The ZPNiIn sensor presents excellent HCHO-sensing properties with a high response value, high rapid response/recovery speed, and excellent anti-humidity compared with the other three sensors. The enhanced sensing mechanism of the ZPNiIn HNF-based sensor was further analyzed, which is related to the high catalytic activity of Pt, the formation of interfacial heterojunction, and the appropriate loading of ZIF-8 nanoparticles.

## 2. Materials and Methods

### 2.1. Chemicals

All the chemicals and reagents used in this work can be found in the Supporting File (Text S1).

### 2.2. Synthesis of Pristine In_2_O_3_ and NiO/In_2_O_3_ Hollow Nanofibers (NiIn HNFs)

NiIn HNFs were prepared with the one-step electrospinning method. In detail, 1 g of In(NO_3_)_3_·4.5H_2_O and 0.2 g of Ni(NO_3_)_2_·6H_2_O were dissolved in N,N-dimethylformamide (8 mL) and ethanol (5 mL) under vigorous stirring. Then, an electrospun precursor was formed by adding 1 g of PVP to the mixture and stirring for 6 h at room temperature. An electrostatic voltage of 14 kV was applied between the needle tip of a 10 mL syringe containing the above precursor. Finally, further calcination at 600 °C for 3 h in air with a heating rate of 5 °C per min^−1^ formed NiIn HNFs from the precursor fibers. This same process was used to prepare the pristine In_2_O_3_ HNFs without Ni(NO_3_)_2_·6H_2_O.

### 2.3. Synthesis of ZIF-8@NiO/In_2_O_3_ (ZNiIn) and ZIF-8@Pt-NiO/In_2_O_3_ (ZPNiIn) HNFs

In the ZIF-8@Pt-NiO/In_2_O_3_ (ZPNiIn) HNF synthesis process, 0.4 g of zinc oxide and 0.6 g of 2-methylimidazole were dissolved in methanol (30 mL), forming mixture A. Then, 0.05 g of as-prepared NiIn HNFs was suspended in methanol with 0.15 mL of 10 mg mL^−1^ H_2_PtCl_6_·6H_2_O solution. The prepared suspension was ultrasonically treated for 20 min; then, a reducing agent of 0.008 g of NaBH_4_ was added dropwise, and the mixture was stirred to form mixture B. The above two solutions were then mixed and continually stirred to form a homogenous solution. Then, the solution was transferred to a Teflon reactor and heated to 120 °C for 3 h. We collected the precipitate and washed it with methanol after centrifugation. Finally, the obtained ZPNiIn HNFs were dried at 60 °C overnight. The ZNiIn HNFs were obtained with the same process without the addition of H_2_PtCl_6_·6H_2_O and NaBH_4_.

### 2.4. Characterizations

SEM (GeminiSEM 500, China) and TEM (JEOL JEM2100, Japan) were used to characterize the samples’ morphology. X-ray diffraction (XRD) patterns were acquired on a PANalytical X’pert MPDPro (The Netherlands) using a Cu Kα radiation source (40 kV, 40 mA). The AXIS ULtrabld (UK) instrument was used to obtain X-ray photoelectron spectroscopy (XPS) using a monochromatic Al Kα radiation source (15 kV, 1486.6 eV). Fourier-transform infrared spectroscopy (Bruker Tensor 37 spectrometer, China) was used to determine surface functional groups and chemical bonds of samples. An HR800 Raman spectrometer (France) was used for Raman scattering, and excitation was carried out with 532 nm laser light. The specific surface area and pore sizes of the samples were detected with N_2_ adsorption/desorption using an SSA-4300 (China) instrument. Sessile drops were measured using the optical contact angle meter (OCA25HTV, DataPhysics, China), where distilled water drops were placed on solid surfaces using a dispenser. 

### 2.5. Gas Sensor Fabrication and Sensing Tests

Using drops of deionized water, the prepared materials were mix into a homogeneous paste. Then, this paste was coated uniformly on the ceramic tube with Au electrodes, further annealed in a muffle furnace, and annealed at 150 °C for 4 h. As shown in Figure S1, gas-sensing performance was evaluated using a WS-30A measurement system after aging the sensors for seven days in air [22]. A static liquid–gas distribution method was used to prepare the different concentrations of tested gases. First, reference air from the cylinder was continuously introduced into the gas distribution chamber at a rate of 50 mL/min to replace the air in the gas distribution chamber for 10 min. After stopping the introduction of reference air, a certain volume Q of HCHO solution (37%) was collected using a microsyringe, injected into the evaporator in the gas distribution chamber, and heated to evaporation. Finally, the HCHO vapor is completely mixed with the reference air using a built-in fan. Specifically, acetone, benzene, toluene, methanol, ethanol, and formaldehyde vapors were obtained by evaporating acetone (≥99.5%), benzene (≥99.5%), toluene (≥99.5%), methanol (≥99.5%), ethanol (≥99.7%), and formaldehyde (37%) solution, respectively, and the methane gas was the standard gas. The volume Q can be obtained with the Equation (S1). The specific description is shown in Text S2.

## 3. Results and Discussion

### 3.1. Morphology Characterization and Phase Composition

**Figure 1:**
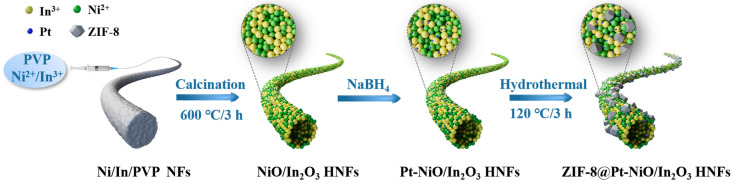
Schematic illustration of the fabrication of ZIF-8@Pt-NiO/In_2_O_3_ hollow nanofibers.

**Figure 2:**
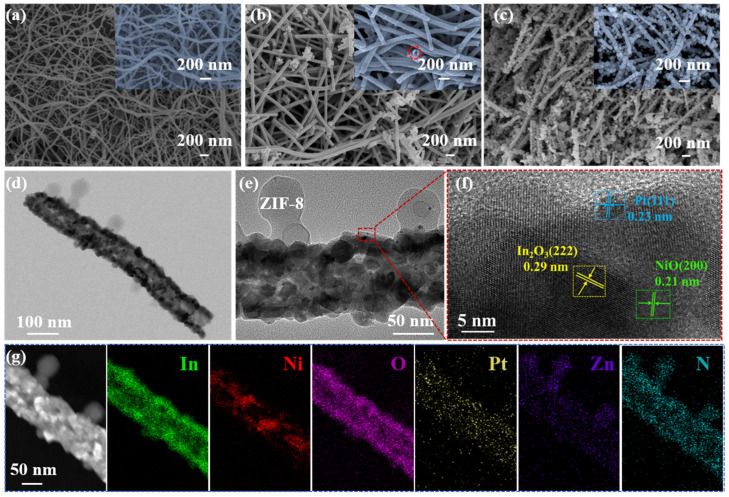
SEM images of as-prepared (**a**) pristine In_2_O_3_ HNFs, (**b**) NiO/In_2_O_3_ HNFs (red circle is the hollow structure), (**c**) ZIF-8@Pt-NiO/In_2_O_3_ HNFs; (**d**,**e**) TEM images, (**f**) HRTEM, and (**g**) EDS element mapping of ZIF-8@Pt-NiO/In_2_O_3_ HNFs.

The schematic illustration of the ZIF-8@Pt-NiO/In_2_O_3_ HNFs is illustrated in Figure 1 [22]. SEM images, as illustrated in Figure 2a–c, present that the obtained nanofibers of pristine In_2_O_3_, NiIn, ZNiIn, and ZPNiIn HNFs consist of continuous nanofibers that form web-like network structures (Figure 2a–c and Figure S2). A clear hollow structure of NiIn nanofibers can be observed in Figure 2b (red circle in inset Figure 2b). Unlike the smooth surfaces of In_2_O_3_ and NiIn HNFs, the ZPNiIn surface was uniformly loaded with ZIF-8 nanoparticles. The sharp contrast occurred between the dark edge and the pale interval in TEM images (Figure 2d,e) of ZIF-8@Pt-NiO/In_2_O_3_, confirming the hollow structure. Lattice fringes of 0.23, 0.29, and 0.21 nm correspond to the crystal planes of Pt (111) [23], In_2_O_3_ (222) [24], and NiO (200) [10], respectively (Figure 2f). The EDS elemental mappings in Figure 2g reveal that In, Ni, O, Pt, Zn, and N elements evenly distribute in ZPNiIn HNFs, proving the formation of a heterojunction. The EDS spectrum shown in Figure S3 further presents that the percentages of In, Ni, O, Pt, Zn, and N are 22.68%, 6.54%, 68.22%, 0.48%, 1.39%, and 0.69%, respectively.

**Figure 3:**
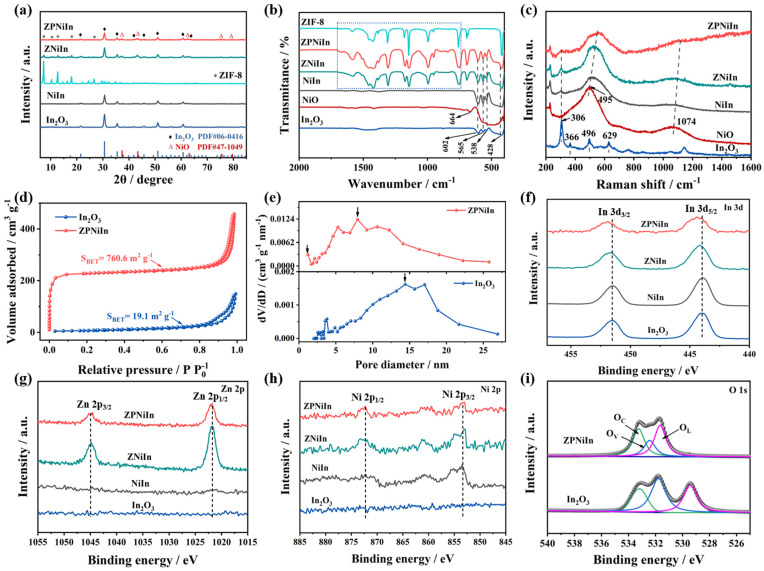
Structural characterizations for pristine In_2_O_3_, NiIn, ZNiIn, and ZPNiIn HNFs. (**a**) XRD patterns; (**b**) FT-IR spectra and (**c**) Raman spectra of various samples. (**d**) N_2_ adsorption–desorption isotherms and (**e**) pore-size distributions of pristine In_2_O_3_ and ZPNiIn HNFs; XPS spectra of (**f**) In 3d, (**g**) Zn 2p and (**h**) Ni 2p of various samples, and (**i**) O 1s of pristine In_2_O_3_ and ZPNiIn HNFs.

The phases and crystallinities of pristine In_2_O_3_, NiIn, ZNiIn, and ZPNiIn HNFs are examined using XRD patterns, as illustrated in Figure 3a. Pristine In_2_O_3_ exhibits a cubic-phase In_2_O_3_ (JCPDS No. 06-0416) [25]. The as-synthesized NiIn nanocomposites are composed of the two phases of In_2_O_3_ and NiO. The diffraction peaks of the XRD spectra of ZNiIn and ZPNiIn HNFs correspond to cubic In_2_O_3_ (JCPDS No. 06-0416), ZIF-8, and NiO (JCPDS No. 47-1049), respectively [26]. No obvious peak belonging to Pt is observed in ZPNiIn HNFs, indicating that adding Pt will not change the crystal structure of NiO/In_2_O_3_ and that the Pt content is too low to detect the XRD signal [27]. The FT-IR spectra are shown in Figure 3b. The spectrum of pristine In_2_O_3_ with distinctive intense bands at 428, 538, 565, and 602 cm^−1^ could be ascribed to the stretching vibrations of the In-O [28]. For the FT-IR curve of ZPNiIn, some new absorption peaks belonging to the stretching vibrations of Ni-O and ZIF-8 are observed [29], suggesting the successful formation of the ZPNiIn HNFs. 

To further investigate the crystal structure of the as-prepared samples, the Raman spectra were investigated. As shown in Figure 3c, the Raman spectrum of pristine In_2_O_3_ presents peaks located at 306, 496, and 629 cm^−1^, which are ascribed to the δ (InO_6_), In-O-In, and υ(InO_6_), respectively [25]. The peak of NiO at 495 cm^−1^ belongs to the optical mode of longitudinal vibration (LO). The peak located at 1074 cm^−1^ indicates the 2LO phonon vibration of NiO [30]. Compared with the In_2_O_3_ and NiO, the Raman peaks of NiIn, ZNiIn, and ZPNiIn HNFs exhibit a slight blue shift due to the electron–phonon interaction generated by heterojunction formation, indicating that heterojunctions can modulate the chemical bonds and electrical properties [31].

The nitrogen adsorption–desorption measurements of pristine In_2_O_3_ and ZPNiIn HNFs were carried out in Figure 3d,e. For the pristine In_2_O_3_ and ZPNiIn HNFs, the nitrogen adsorption/desorption isotherms show the typical IV isotherm with an H3 hysteretic loop, indicating the coexistence of micropores and mesopores (Figure 3d). Furthermore, the filling of ZIF-8 micropores increased ZPNiIn HNF sorption under low relative pressure [32]. The BET surface area of the ZPNiIn HNFs is 760.6 m^2^ g^−1^, which is a nearly 40-fold increase compared to that of pristine In_2_O_3_ HNFs (19.1 m^2^ g^−1^), demonstrating the enhanced gas adsorption performance of ZPNiIn HNFs. The average pore diameter of pristine In_2_O_3_ and ZPNiIn HNFs are 7.9 nm and 23.9 nm, respectively. 

The XPS spectra of samples were obtained to analyze the elemental composition and valence states. The peaks at 443.9–444.3 eV and 451.5–451.9 eV are found in all the samples corresponding to In 3d_5/2_ and In 3d_3/2_, respectively, verifying the presence of In^3+^ [33] (Figure 3f). For Zn 2p spectra, peaks corresponding to Zn 2p_3/2_ and Zn 2p_1/2_ of Zn^2+^ at 1021.8 eV and 1044.7 eV are observed in ZNiIn and ZPNiIn HNFs due to the ZIF-8 loaded on the surface of NiO/In_2_O_3_ HNFs (Figure 3g). The spectra of Ni 2p (Figure 3h) present two peaks of Ni 2p_1/2_ (871.6 eV) and Ni 2p_3/2_ (853.1 eV) for NiIn, ZNiIn, and ZPNiIn samples, indicating the existence of Ni^2+^ [34]. Figure 3i shows the O 1s spectra of pristine In_2_O_3_ and ZPNiIn HNFs. The spectra of O 1s can be categorized as lattice oxygen (O_L_), oxygen vacancies (O_V_), and chemisorbed oxygen (O_C_) [35]. The area ratios of the oxygen fractions of samples are further listed in Table S1. The adsorption of air oxygen on the surface of the sensing materials results in the formation of O_C_, which can react with the analyzed gas [36]. The contents of O_C_ in pristine In_2_O_3_ and ZPNiIn HNFs are 23.3% and 36.9%, demonstrating the ZIF-8 loaded and the heterojunction formation contribute to increasing the surface O_C_ content. The significantly increased proportion of O_C_ can facilitate the gas-sensing reaction and enhance the sensitivity. 

### 3.2. Gas-Sensing Performance

**Figure 4:**
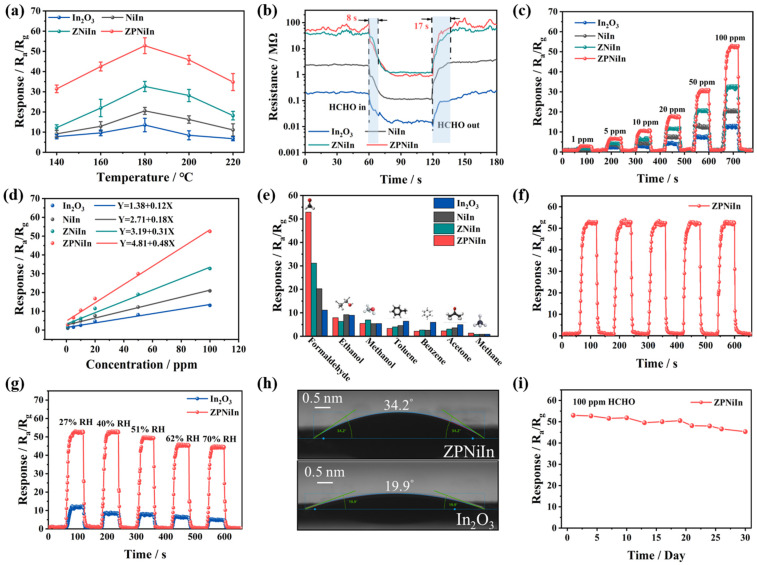
Sensing property measurements for pristine In_2_O_3_, NiIn, ZNiIn, and ZPNiIn HNFs at 180 °C. (**a**) Response toward HCHO (100 ppm) under different operating temperatures; (**b**) response and recovery characteristics of sensor exposure to HCHO (100 ppm); (**c**) response curves of sensors to HCHO from 1 to 100 ppm concentrations; (**d**) linearity between the concentration of sensors and the response value; (**e**) selectivity of sensors toward 100 ppm of various VOC gas; (**f**) repeatability of the ZPNiIn sensor to HCHO (100 ppm); (**g**) dynamic response curves of the pristine In_2_O_3_ and ZPNiIn sensors to HCHO (100 ppm) under different relative humidities (RHs); (**h**) water contact angles of the pristine In_2_O_3_ and ZPNiIn HNF-sensing materials’ surface; (**i**) long-term stability of ZPNiIn sensor towards HCHO (100 ppm).

To evaluate the effect of p-n heterojunctions, ZIF-8, and noble metal Pt nanoparticles on the sensor performance, HCHO-sensing measurements based on pristine In_2_O_3_, NiIn, ZNiIn, and ZPNiIn sensors were carried out. As depicted in Figure 4a, the responses of pristine In_2_O_3_, NiIn, ZNiIn, and ZPNiIn HNF sensors to HCHO (100 ppm) were investigated at the operating temperatures of 140–220 °C. The results of the four sensors indicate that response values are trending upward until they reach the maximum of 13.5 (In_2_O_3_), 20.5 (NiIn), 32.6 (ZNiIn), and 52.8 (ZPNiIn) at 180 °C. Then, the responses decreased as the operating temperature increased from 180 to 220 °C. Among them, the ZPNiIn sensor exhibits an excellent response value, 3.9-fold higher than the In_2_O_3_ sensor. The substantial enhancement in the HCHO response value can be ascribed to the synergistic impact of ZIF-8 and Pt modification and the construction of a heterojunction. Hence, the optimal operating temperature for the sensors is 180 °C. Figure S4 displays the baseline sensor resistance (R_a_) in air at the operating temperatures of 140–220 °C. The results reveal that the R_a_ of the four sensors decreases with increasing temperature, which is due to the characteristics of metal oxide semiconductors [37]. Notably, the resistance of ZNiIn and ZPNiIn sensors dramatically increased, which was caused by the poor electric properties of ZIF-8 [38].

Figure 4b and Figure S5 depict the real-time response and recovery curves of four sensors toward 100 ppm HCHO at 180 °C. The response/recovery time of pristine In_2_O_3_, NiIn, ZNiIn, and ZPNiIn sensors are 15/34 s, 10/12 s, 10/14 s, and 8/17 s, respectively. Compared to pristine In_2_O_3_, the ZPNiIn sensor offers a 2-fold increase in response/recovery speed. Figure 4c reveals the gas response curves of sensors toward different HCHO concentrations (1–100 ppm) at 180 °C. It can be seen from the curve that the gas response values of sensors increase as the HCHO concentration increased. Among these four sensors, the ZPNiIn sensor presents the highest response values toward different concentrations of HCHO. Moreover, all sensors present good linearity relationship between HCHO concentrations and the response within the given concentration range (Figure 4d). The theoretic limits of detection (*LODs*) of the sensor can be calculated based on Equation (1) [39,40]:

(1) $$LOD=\frac{3\times {RMS}_{noise}}{slope}$$

where *RMS_noise_* denotes the root mean square noise based on 50 experimental baselines in the air. The *slope* can be obtained in the concentration–response curve. After calculation, the LOD of the ZPNiIn sensor to HCHO gas is calculated as low as 46.7 ppb, while the LODs of pristine In_2_O_3_, NiIn, and ZNiIn are 258.2, 122.3, and 63.8 ppb, respectively, implying the ZPNiIn sensor can be used to detect HCHO at lower ppb concentrations.

Furthermore, the responses of four sensors toward different interfered volatile organic compound (VOC) gases (100 ppm) were also investigated at 180 °C (Figure 4e). The response of the ZPNiIn sensor to HCHO is significantly higher than to other interfering gases. Meanwhile, the sensitivity of the ZPNiIn sensor surpasses that of other sensors, demonstrating its excellent selectivity. The five consecutive cyclic response/recovery curves of the ZPNiIn sensor toward 100 ppm HCHO at 180 °C are shown in Figure 4f and Figure S7, and the response value of the sensors only fluctuates slightly with unchanged response/recovery times, suggesting the excellent reproducibility of the ZPNiIn sensor.

The interference of humidity on pristine In_2_O_3_ and ZPNiIn sensors’ sensitivity toward 100 ppm HCHO was also evaluated as displayed in Figure 4g. With the increase in humidity from 27% to 70% RH, the HCHO response value of the ZPNiIn sensor is reduced by 14.2%, which is significantly superior to that of the pristine In_2_O_3_ sensor (the response decreased by 58.7%). Meanwhile, the baseline resistances of both sensors are decreased with increasing humidity (Figure S6). Notably, the ZPNiIn sensor can still achieve a high response value to HCHO under 70% RH (R_a_/R_g_ = 44.4), indicating the improved humidity tolerance of the sensor. The water contact angles between sensing materials and drops of water are shown in Figure 4h. The ZPNiIn has stronger hydrophobicity with the water contact angle of 34.2°, which is bigger than pristine In_2_O_3_ (19.9°). The improved anti-humidity of the ZPNiIn sensor is mainly attributed to the surface modifications with ZIF-8 coating [41] and the functionalization of Pt noble metals [15,42]. Based on Figure 4i, the sensitivity of the ZPNiIn sensor toward HCHO has barely fluctuated over 30 days, implying the significant long-term stability of the ZPNiIn sensor. To further estimate the sensing properties of the ZPNiIn sensor, some similar works on HCHO gas-sensing are summarized as listed in Table S2. The ZPNiIn sensor also exhibits a more outstanding sensitivity toward HCHO gas compared with those reported HCHO sensors. 

### 3.3. Gas-Sensing Mechanism

During the adsorption and desorption of target gas molecules on the surface of the sensing material, MOS changes its electrical resistance to detect the gas [43,44]. The obtained ZIF-8@Pt/NiO-In_2_O_3_ presented n-type semiconducting gas-sensing characteristics. In the ambient atmosphere, the adsorbed oxygen on the sensing material surface is then ionized into $O_{2}^{-}$, O^−^, or O^2−^, depending on when the operating temperature is 150 °C or less, between 150 and 400 °C, or more than 400 °C, respectively [45]. The optimum temperature in our work is 180 °C. Thus, the adsorbed oxygen ions of this work are O^−^ (Equation (3)). Once the HCHO gas has been introduced, it reacts with O^−^, resulting in the generation of CO_2_ and electrons (Equation (4)); electrons are released again into the sensing material’s conduction band. The process is expressed by Equations (2)–(4) as follows:

O_2_ (gas) → O_2_ (ads) (2)

O_2_ (ads) + e^−^ → 2O^−^ (ads) (3)

HCHO (gas) +2O^−^ (ads) → CO_2_ (gas) + H_2_O (gas) + 2e^−^ (4)

**Figure 5:**
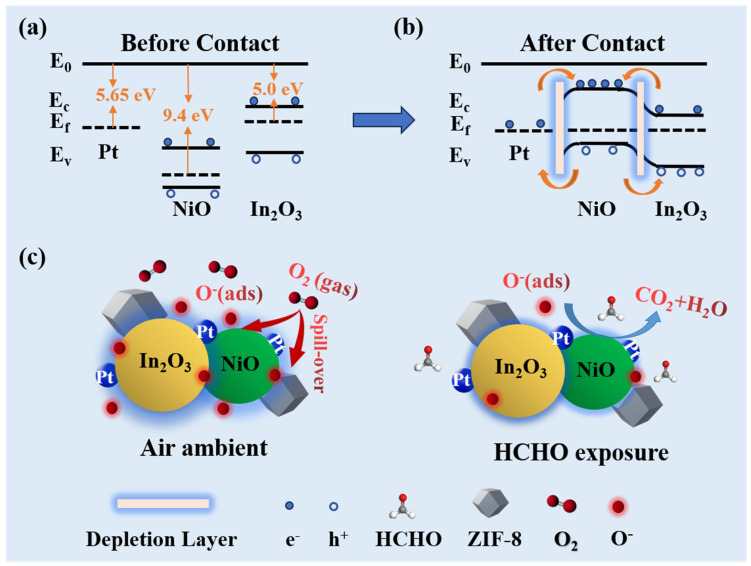
(**a**,**b**) The energy level diagram and (**c**) the schematic sensing mechanism of ZPNiIn HNFs.

Figure 5 displays the schematic diagram of ZPNiIn for HCHO gas sensing. Sensors based on ZPNiIn are more sensitive to HCHO than those based on other materials. This could be explained as follows:

On the one hand, heterojunctions increase oxygen molecule absorption and target gas detection. When these three components come in contact, since the work function of NiO (9.4 eV) [37] is greater than that of In_2_O_3_ and Pt, electrons will migrate from In_2_O_3_ and Pt to NiO until their Fermi energy levels reach equilibrium, and the Schottky barrier formed at the contact interfaces, as shown in Figure 5a,b. The aggregation of electrons on NiO increases its concentration and improves its ability to capture oxygen, thereby promoting oxygen adsorption. This results in the superior performance of the isopropanol sensor since the resistance changes considerably during the sensing process.

On the other hand, the chemical sensitization of Pt NPs on the surface of NiO/In_2_O_3_ composites is the main reason for the improvement of the gas-sensing capability [46,47]. The Pt NPs facilitate the dissociation of O_2_ molecules into adsorbed oxygen (O^−^) through the “spillover effect”, increasing the concentration of adsorbed oxygen (Figure 5c). Therefore, ZPNiIn possessed more surface-adsorbed oxygen, which is favorable to promote the active sites for enhancing the gas-sensing properties [48]. The characterization results of XPS also confirmed that the adsorbed oxygen content of ZPNiIn increased (Table S1). 

Moreover, the coated ZIF-8 NPs also play a significant role in the improvement of the sensing capabilities. From the results of the N_2_ adsorption–desorption measurement, the specific surface area of the ZPNiIn HNFs is increased 40-fold due to the load of ZIF-8. This can increase the number of active sites available for the adsorption of the target HCHO gas during sensing [38,49]. For practical applications, water vapor is partially repellent from the hydrophobic ZIF-8 and does not interfere with sensing performance under humidity conditions [50]. In addition, gas adsorption and diffusion are facilitated by the inner and outer surfaces of ZPNiIn hollow structures, which provide ample active sites for gas reactions [51]. 

## 4. Conclusions

In order to develop a high-performance HCHO sensor for indoor pollutant detection, the ZIF-8 loaded Pt/NiO-In_2_O_3_ HNFs were synthesized using the electrospinning method combined with hydrothermal techniques. The ZPNiIn sensor exhibits an enhanced response (R_a_/R_g_) of 52.8 towards 100 ppm HCHO with a fast response (8 s) and recovery (17 s) time at 180 °C. Moreover, the sensor shows excellent selectivity and good anti-humidity. The improved gas-sensing properties can be attributed to the heterojunctions between components, the catalytic and sensitization effects of Pt NPs, and the coated ZIF-8. This study presents an effective strategy to design and fabricate high-performance HCHO sensors.

## Data Availability

The data that have been used are confidential.

## Supplementary Materials

The following supporting information can be downloaded at: https://www.mdpi.com/article/10.3390/nano14100841/s1, Figure S1. Photographic images and schematic diagram of a fabricated gas sensor and WS-30A measurement system; Figure S2: SEM image of as-prepared ZNiIn HNFs; Figure S3: EDS spectrum for ZPNiIn HNFs; Figure S4: Base resistance in air of all sensors under different operating temperatures; Figure S5: Response–recovery curves of four sensors toward 100 ppm HCHO at 180 °C; Figure S6: The baseline resistances of pristine In_2_O_3_ and ZPNiIn sensors in the air under different relative humidity conditions; Figure S7: The repeatability of four parallel sensors based on ZPNiIn HNFs to HCHO (100 ppm); Table S1: The relative percentages of three different oxygen species for pristine In_2_O_3_, and ZPNiIn HNFs; Table S2: Comparison of HCHO gas-sensing performance with other gas sensors. References [39,52,53,54,55] are cited in the Supplementary Materials.
