# Supplementary material for: Synergistic Effect of ZIF-8 and Pt-Functionalized NiO/In2O3 Hollow Nanofibers for Highly Sensitive Detection of Formaldehyde
Source: Nanomaterials (Basel). 2024 May 10;14(10):841. doi: 10.3390/nano14100841 (PMC11124443; doi:10.3390/nano14100841)
Supplement: Supplementary file 1 [file nanomaterials-14-00841-s001.zip › nanomaterials-2980658-supplementary.pdf]

Supporting Information

Synergistic Effect of ZIF-8 and Pt-functionalized NiO/In_2O_3 Hollow Nanofibers for Highly Sensitive Detection of Formaldehyde

Lei Zhu [1,2,†], Ze Wang [1,†], Jianan Wang [1*], Jianwei Liu [3,4], Wei Zhao [2], Jiaxin Zhang [1], and Wei Yan [1,*]

[1] Xi'an Key Laboratory of Solid Waste Resource Regeneration and Recycling, State Key Laboratory of Multiphase Flow Engineering, School of Energy and Power Engineering, Xi'an Jiaotong University, Xi'an 710049, China
[2] School of Physics and Electrical Engineering, Weinan Normal University, Chaoyang Street, Weinan 714099, China
[3] Xianggui Manganese Industry Co., Ltd. Ziyang, Ankang 725300, China
[4] School of Chemistry and Chemical Engineering, Xi'an University of Science & Technology, Xi'an 710054, China
* Correspondence: wangjn116@xjtu.edu.cn (Jianan Wang); yanwei@xjtu.edu.cn (Wei Yan).
† These authors contributed equally to this work

Text. S1 Chemicals

Indium nitrate hydrate ($In(NO_3)_3 \cdot 4.5H_2O$) and nichel nitrate hexahydrate ($Ni(NO_3)_2 \cdot 6H_2O$) were purchased from Macklin Biochemical Technology Co., Ltd., China. Zinc nitrate hexahydrate ($Zn(NO_3)_2 \cdot 6H_2O$), poly (vinylpyrrolidone) (PVP, Mw=1.3×10^6), chloroplatinic acid hexahydrate ($H_2PtCl_6 \cdot 6H_2O$), ethanol (EtOH) (99.0%), N, N- dimethylformamide (DMF), and 2-methylimidazole (2-MeIM) were used as received by Sinopharm Chemical Reagent Co. Ltd., China. All chemicals were used without further purification.

Text. S2

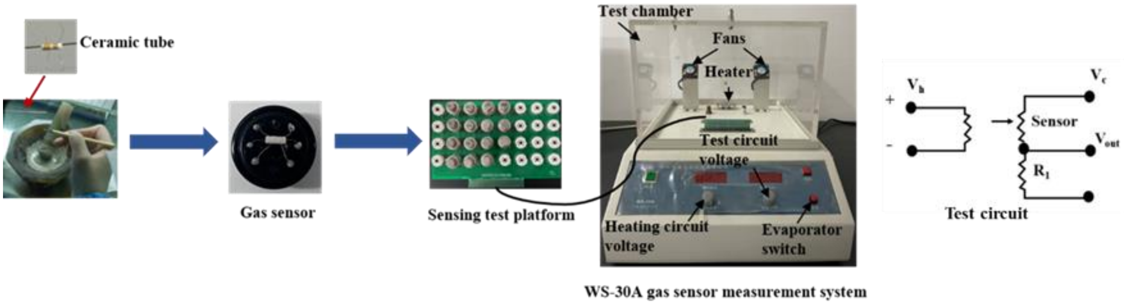

Fig. S1. Photographic images and schematic diagram of a fabricated gas sensor and WS-30A measurement system.

The photographic images and schematic diagram of the fabricated sensor and WS-30A measurement system were displayed in Fig. S1. The volume of gas distribution chamber was 18 L. The measurement power (V_c) was 1.5-10 V. The operating temperature of the sensor was controlled by regulating the voltage of the heating wire. The different concentrations of test gases were prepared based on the static gas distribution method. A certain volume Q of gas or liquid was injected into a testing chamber, and the evaporation system quickly volatilized the injected liquid into vapor. The volume Q can be determined by the following equation:

$$Q = \frac{V \times C \times M}{22.4 \times d \times \rho} \times 10^{-9} \times \frac{273 + T_R}{273 + T_B} \quad \text{(S1)}$$

Where V, C, M, d, ρ, T_R, and T_B are the test chamber volume (mL), vapor concentration (ppm), liquid molecular mass (g), liquid density (g /cm^3), liquid purity, environmental temperature (°C), and temperature in the testing chamber (°C), respectively. The stable resistance value of the sensor in air (or test gas) is labeled as R_a (or R_g). The sensitivity is defined as $S = R_a/R_g$. The response and recovery times are calculated based on the time from its response to reach 90% variation of the total resistance.

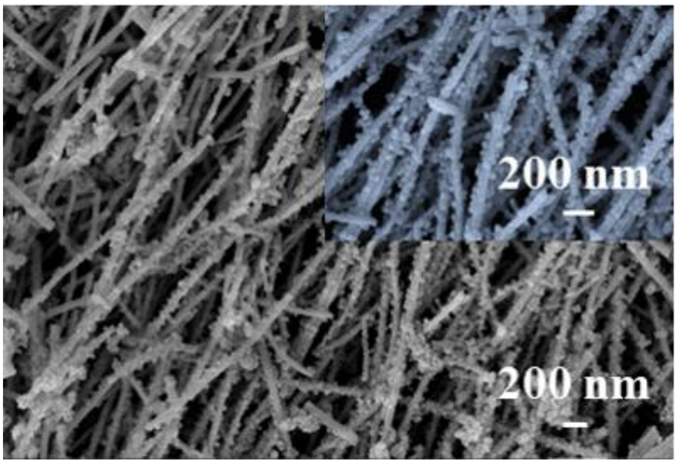

Figure. S2 SEM image of as-prepared ZNiIn HNFs

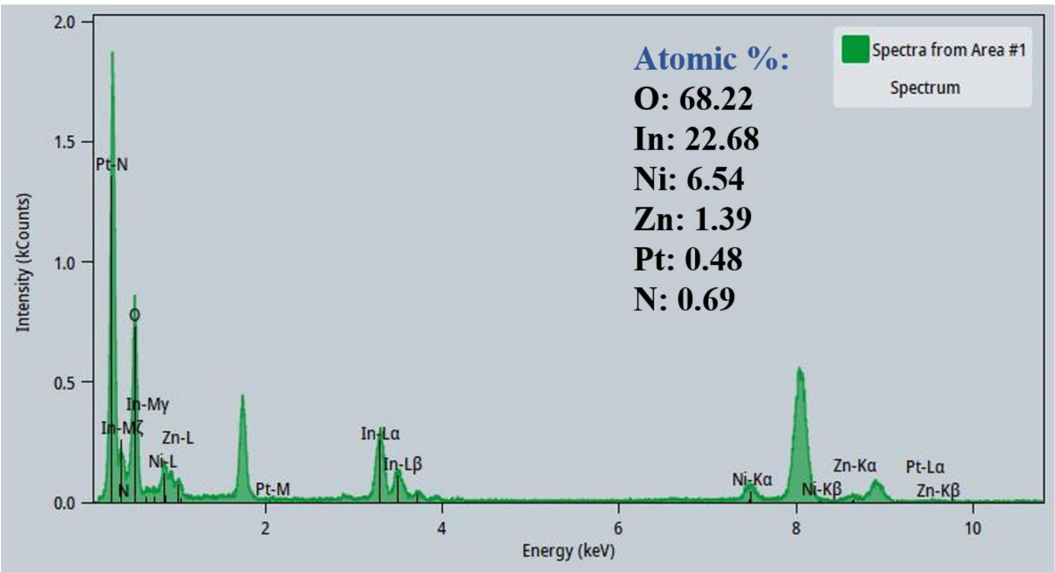

Figure. S3 EDS spectrum for ZPNiIn HNFs

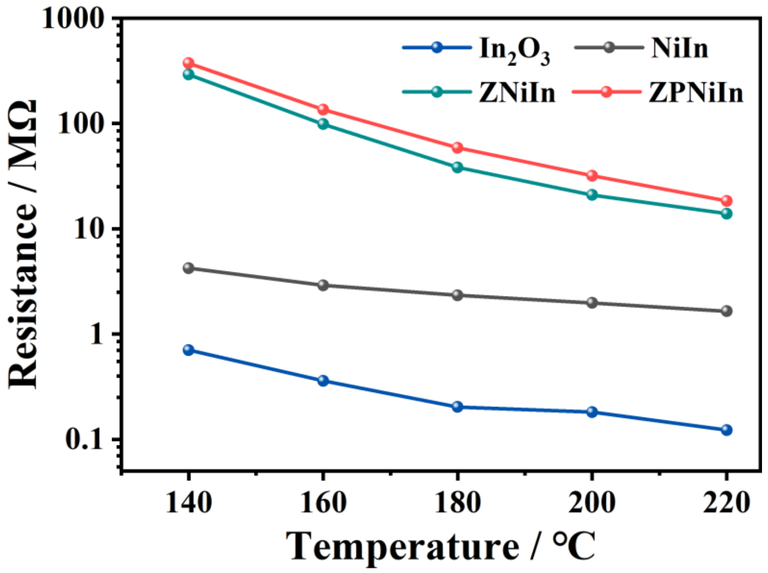

Figure. S4 Base resistance in air of all sensors under different operating temperatures.

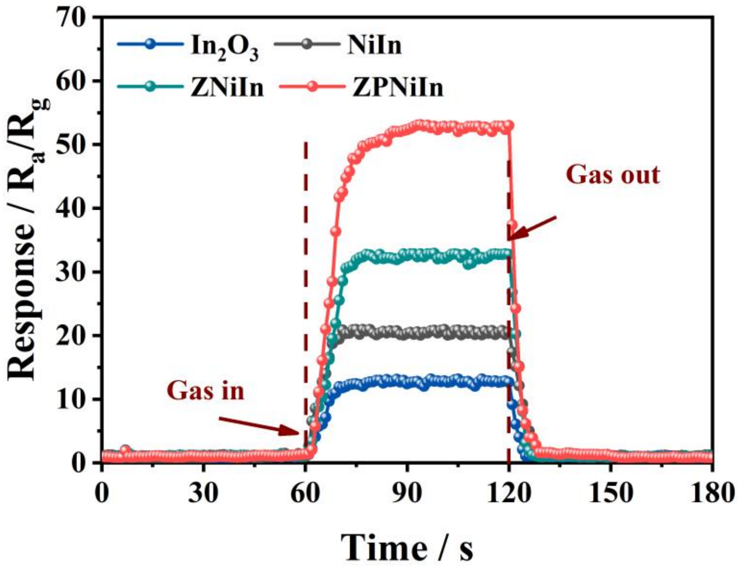

Figure. S5 Response-recovery curves of four sensors toward 100 ppm HCHO at 180 ℃.

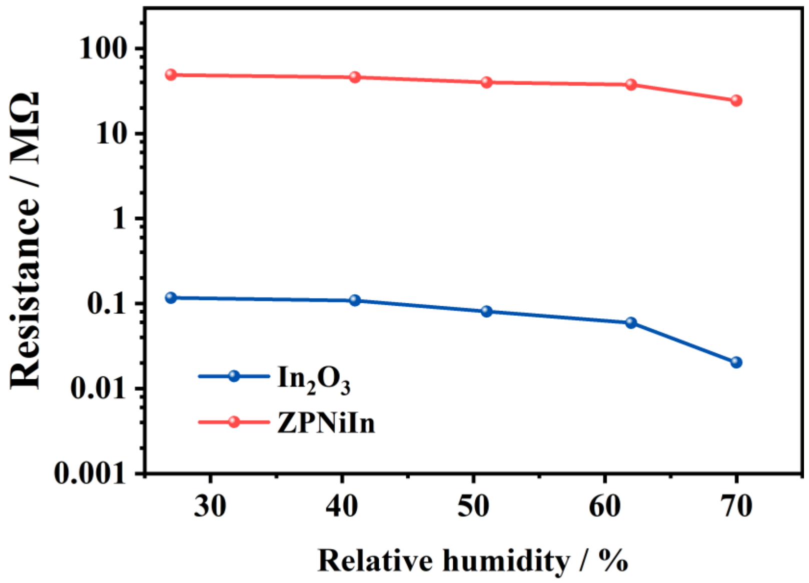

Figure. S6 The baseline resistances of pristine In_2O_3 and ZPNiIn sensors in the air under different relative humidity conditions.

The baseline resistances of pristine In_2O_3 and ZPNiIn sensors are decreased with the increased humidity (Figure. S6). This phenomenon is mainly attributed to the water molecules adsorbed on the surface of sensing material to form more charge carriers, which is conducive to electrical conduction.

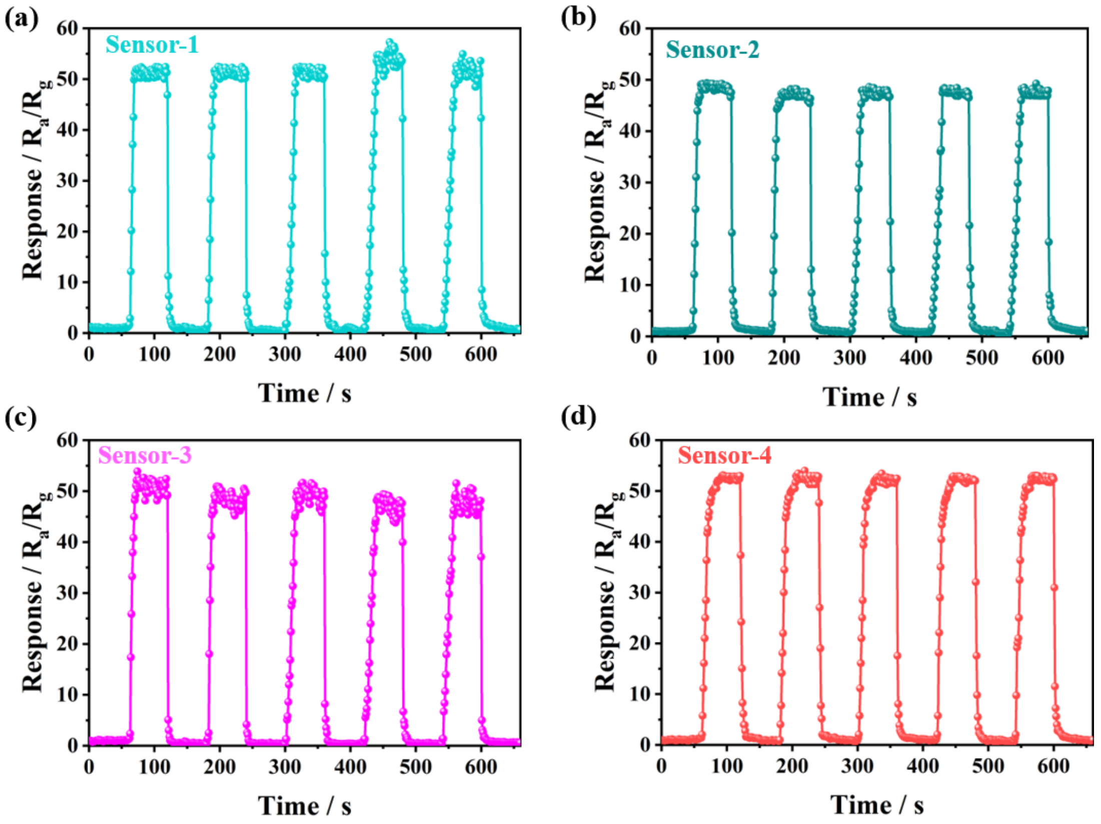

Figure S7 The repeatability of four parallel sensors based on ZPNiIn HNFs to HCHO (100 ppm)

As shown in Figure S7, the responses of four parallel sensors based on ZPNiIn HNFs only fluctuates slightly with unchanged response/ recovery times, suggesting the excellent repeatability and reproducibility of the ZPNiIn sensors. And The response values of the four parallel sensors for HCHO ranged from 46-53, with no more than 13% difference between the response values.

Table S1. The relative percentages of three different oxygen species for pristine In_2O_3, and ZPNiIn HNFs.

Sample	Oxygen Species	Relative Percentage (%)
pristine In_2O_3	O_L	32.5
	O_v	44.2
	O_c	23.3
ZPNiIn	O_L	41.5
	O_v	21.6
	O_c	36.9

Table S2. Comparison of HCHO gas sensing performance with other gas sensors.

Materials	Temperature / ℃	Concentration / ppm	Response	t_{res}/t_{rec} / s	Ref.
SnO_2/Fe_2O_3	220	20	4.5	9 / 34	[1]
In_2O_3/Co_3O_4	180	100	15.7	32 / 42	[2]
Au-In_2O_3	240	100	37	3 / 8	[3]
SnO_2	200	100	38.3	17 / 25	[4]
Er/In_2O_3	260	20	12	5 / 38	[5]
ZPNiIn HNFs	180	100	52.8	8 / 17	**This work**

References

1. Lou, C.; Huang, Q.; Li, Z.; Lei, G.; Liu, X.; Zhang, J. Fe_2O_3-sensitized SnO_2 nanosheets via atomic layer deposition for sensitive formaldehyde detection. *Sens. Actuators, B* **2021**, *345*, doi:10.1016/j.snb.2021.130429.
2. Cao, J.; Zhang, N.R.; Wang, S.M.; Zhang, H.M. Electronic structure-dependent formaldehyde gas sensing performance of the In_2O_3/Co_3O_4 core/shell hierarchical heterostructure sensors. *J. Colloid Interface Sci.* **2020**, *577*, 19-28, doi:10.1016/j.jcis.2020.05.028.
3. Zhang, S.; Song, P.; Li, J.; Zhang, J.; Yang, Z.; Wang, Q. Facile approach to prepare hierarchical Au-loaded In_2O_3 porous nanocubes and their enhanced sensing performance towards formaldehyde. *Sens. Actuators, B* **2017**, *241*, 1130-1138, doi:10.1016/j.snb.2016.10.023.
4. Li, Y.; Chen, N.; Deng, D.; Xing, X.; Xiao, X.; Wang, Y. Formaldehyde detection: SnO_2 microspheres for formaldehyde gas sensor with high sensitivity, fast response/recovery and good selectivity. *Sens. Actuators, B* **2017**, *238*, 264-273, doi:10.1016/j.snb.2016.07.051.
5. Wang, X.S.; Zhang, J.B.; Wang, L.Y.; Li, S.C.; Liu, L.; Su, C.; Liu, L.L. High response gas sensors for formaldehyde based on Er-doped In_2O_3 nanotubes. *Journal of Materials Science & Technology* **2015**, *31*, 1175-1180, doi:10.1016/j.jmst.2015.11.002.
